# Bridging the Gap: Improving Locum Rates at South West London and St George’s Mental Health NHS Trust

**DOI:** 10.1192/bjo.2025.10353

**Published:** 2025-06-20

**Authors:** Tamara Clarke

**Affiliations:** South West London and St George’s Mental Health NHS Trust, London, United Kingdom

## Abstract

**Aims:** The 2023 GMC national trainee survey revealed that 33% of secondary care trainers reported that their trainees’ education and training were adversely affected because rota gaps aren’t always dealt with appropriately. This project aimed to improve locum rates within the South West London and St George’s Mental Health NHS Trust (SWLSTG) to address these issues and enhance training conditions.

**Methods:** A Freedom of Information (FOI) request was sent to all London mental health trusts to gather data on locum rates at core trainee and registrar levels, escalation policies, and definitions of social and unsocial hours. The data was compiled and compared in a spreadsheet, then presented at the Medical Out of Hours Working Group (MOOHWG) meeting. A new policy was developed to amend out-of-hours pay for doctors based on the findings. This was presented in an executive meeting where it was approved, with changes implemented in August 2024. The process occurred from November 2023–July 2024.

**Results:** The FOI responses revealed that SWLSTG offered less favourable locum rates compared with other London mental health trusts. To bring SWLSTG in line with the local trusts, several key changes were made. The definition of unsocial hours was updated from 9pm–9:30am to the London consensus definition of 7pm–9:30 am on weekdays, as well as all day during weekends and bank holidays. An escalation policy was introduced for shifts first announced with less than 48 hours’ notice, offering a 20% rate increase. Locum rates were also revised: CT1/2 social rate was increased from £40 to £45 per hour, and the unsocial rate from £45 to £54 per hour. CT3 rates were differentiated from CT1/2, with the social rate rising from £40 to £49.25 per hour, and the unsocial rate from £45 to £59.10 per hour. Additionally, the ST4–6 social rate was raised from £45 to £49.25 per hour, and the unsocial rate from £55 to £59.10 per hour.

**Conclusion:** The changes to locum rates and the introduction of an escalation policy at SWLSTG have successfully brought the trust in line with other London mental health trusts. These improvements are expected to reduce the negative impact of rota gaps on trainee education and training, helping to maintain high-quality service delivery and ensure more favourable working conditions for resident doctors. Further evaluation is recommended to assess the long-term impact of these changes on both trainee satisfaction and patient care.

